# Approaching Sex Differences in Cardiovascular Non-Coding RNA Research

**DOI:** 10.3390/ijms21144890

**Published:** 2020-07-10

**Authors:** Amela Jusic, Antonio Salgado-Somoza, Ana B. Paes, Francesca Maria Stefanizzi, Núria Martínez-Alarcón, Florence Pinet, Fabio Martelli, Yvan Devaux, Emma Louise Robinson, Susana Novella

**Affiliations:** 1 Department of Biology, Faculty of Natural Sciences and Mathematics, University of Tuzla, 75000 Tuzla, Bosnia and Herzegovina; amela.jusic@untz.ba; 2 Cardiovascular Research Unit, Department of Population Health, Luxembourg Institute of Health, L-1445 Strassen, Luxembourg; ant.salgado@hotmail.com (A.S.-S.); francescamaria.stefanizzi@lih.lu (F.M.S.); yvan.devaux@lih.lu (Y.D.); 3 INCLIVA Biomedical Research Institute, Menéndez Pelayo 4 Accesorio, 46010 Valencia, Spain; anapaesmarti@gmail.com (A.B.P.); nuriamartinezal96@gmail.com (N.M.-A.); 4 INSERM, CHU Lille, Institut Pasteur de Lille, University of Lille, U1167 F-59000 Lille, France; florence.pinet@pasteur-lille.fr; 5 Molecular Cardiology Laboratory, Policlinico San Donato IRCCS, San Donato Milanese, 20097 Milan, Italy; fabio.martelli@grupposandonato.it; 6 Department of Cardiology, Cardiovascular Research Institute Maastricht (CARIM), Maastricht University, 6229 ER Maastricht, The Netherlands; emma.l.robinson7@googlemail.com; 7 Department of Physiology, Faculty of Medicine and Dentistry, University of Valencia, and INCLIVA Biomedical Research Institute, Menéndez Pelayo 4 Accesorio, 46010 Valencia, Spain

**Keywords:** ncRNA, miRNA, lncRNA, cardiovascular diseases, experimental models, vascular cells, estrogen, androgen, receptors

## Abstract

Cardiovascular disease (CVD) is the biggest cause of sickness and mortality worldwide in both males and females. Clinical statistics demonstrate clear sex differences in risk, prevalence, mortality rates, and response to treatment for different entities of CVD. The reason for this remains poorly understood. Non-coding RNAs (ncRNAs) are emerging as key mediators and biomarkers of CVD. Similarly, current knowledge on differential regulation, expression, and pathology-associated function of ncRNAs between sexes is minimal. Here, we provide a state-of-the-art overview of what is known on sex differences in ncRNA research in CVD as well as discussing the contributing biological factors to this sex dimorphism including genetic and epigenetic factors and sex hormone regulation of transcription. We then focus on the experimental models of CVD and their use in translational ncRNA research in the cardiovascular field. In particular, we want to highlight the importance of considering sex of the cellular and pre-clinical models in clinical studies in ncRNA research and to carefully consider the appropriate experimental models most applicable to human patient populations. Moreover, we aim to identify sex-specific targets for treatment and diagnosis for the biggest socioeconomic health problem globally.

## 1. Introduction

Cardiovascular disease (CVD) is the leading cause of death in females worldwide. Despite this, females are under-represented in clinical trials as well as pre-clinical investigations being the analysis and results rarely stratified according to sex. While males and females share most of the traditional cardiovascular risk factors, their impact on health differs in a sex-specific manner. Although there are clear risk factors unique to females—such as those related to pregnancy, for instance hypertensive pregnancy disorders, preterm delivery, or gestational diabetes—this accounts for a minimal fraction of the mortality and morbidity rates for CVD in females [1].

The risk of developing different entities of CVD shows a sex-related distribution. Heart failure and stroke are two typical examples, with a higher risk in young males compared to females, but shifting to a higher incidence for female individuals after menopause [2,3]. Female and male humans, animals and cells are biologically different. Appropriately equal participation and sex-specific analysis is largely neglected introducing bias in translational findings, clinical concepts, and drug development. Sex differences suggest that biological information, learned from the study of males, may not apply equally to females [4]. Fortunately, the focus of translational biomedical science is changing and new policies have been implemented for the management of patients according to their sex [5]. Recommendations relating to the inclusion of females in research have now been extended beyond clinical research to include cells and animal models [6].

**Figure 1:**
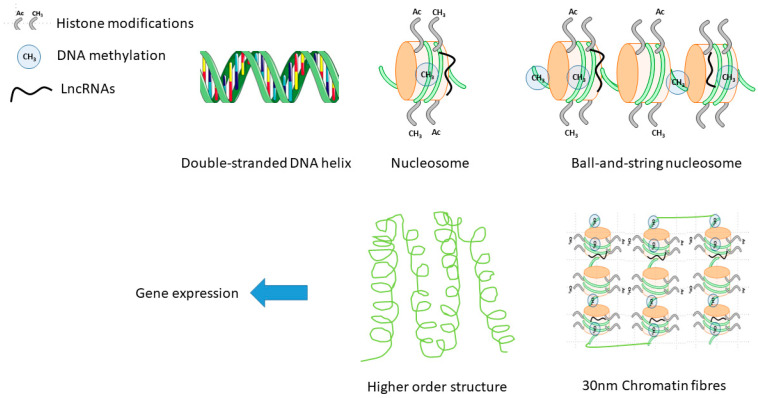
Epigenetic mechanisms involved in regulation of gene expression at the level of transcription.

Epigenetic mechanisms underlie remodeling of human physiology and pathophysiology by tightly regulating gene expression in all cells. Epigenetic regulatory mechanisms include DNA methylation, histone modifications, and non-coding RNAs (ncRNA) and play a pivotal role in cardiovascular differentiation, development, homeostasis, and disease pathophysiology [7] (Figure 1). Sex differences in ncRNA expression profiles, their relationship to disease progression, biomarker value for CVD diagnosis and prognosis as well as clinical outcome are all still poorly understood. Moreover, including both females and males in experimental models of CVD for research is rarely performed. Yet, approaching translational research with sex differences in mind is needed for comprehensive understanding of the sexual dimorphism, which may lead to the improvement of clinical outcome by developing sex-specific diagnostic and therapeutic guidelines. Here, we will review the reasons for the importance of including male and female models for experimental cardiology, the current approaches implemented, and the gaps and inadequate management of sex differences in studying ncRNAs in CVD.

With sex differences emerging in risk, prevalence, sensitivity of biomarkers, and effectivity of treatment options, it formed one of the main topics of the EU-CardioRNA COST Action 3rd working group meeting in 2019 [8]. A roundtable discussion took place, chaired by Susana Novella and Blanche Schroen and this review presents aspects of the discussion of this important topic area.

## 2. Sex-Differences in Cardiovascular Susceptibility

Growing evidence from basic and clinical research demonstrate that sex can affect significantly in susceptibility to CVD. At the biological level, the mechanisms underlying sex differences may include genetic and epigenetic factors, as well as hormonal status.

### 2.1. Role of Chromosomes in Sex-Differences in CVD

In mammals, the presence of a XX or XY pair of chromosomes determines the sexual dimorphism. The genetic information contained in these chromosomes plays a role not only in sexual development but also in the different susceptibility of males and females to complex diseases, such as CVD [9]. Even though sex is a variable present in the adjustment of most multivariable analysis in clinical studies, the sexual chromosomes were historically excluded in experimental designs of genome-wide association studies (GWAS) [9,10]. Nowadays, advanced statistical methods contribute to increasing the knowledge on how haplotypes and polymorphisms associate to relevant phenotypes, outcomes, or treatment response, in a sex-specific manner.

In the context of CVD, an example of sex differences appeared in the study of a cohort of patients with symptomatic aortic stenosis [11]. In this cohort, males and females showed differential left ventricular remodeling. Underlying this difference were certain haplotypes of the chymase 1 (CMA1) gene, which were associated with the left ventricular mass index in males only [11]. Of note, the chymase family is involved in the conversion of angiotensin I into angiotensin II, hence contributing to hypertension [12]. Also, CMA1 activates the TGF-β pathway involved in the pathogenesis of fibrotic and hypertrophic remodeling following pressure overload of the heart [13].

Hypertension is a comorbidity and a serious cardiovascular risk factor contributing to the development of CVD, and its incidence is higher in males than pre-menopausal females [14]. This observation is common for other comorbidities, like the higher prevalence of total cholesterol in females [15]. Male-specific phenotypes can be explained in part by Y chromosome-encoded genes. In the early 1990s, rat models shed light on the link between the Y chromosome and high blood pressure in males [16]. Interestingly, depending on the rat strain used to carry out the experiments, the link between hypertension was either present (in SHR and SHRSP strains) or absent (in SHR/crl strain) [17]. In humans, hypertension seems to have a link to the Y chromosome as it is linked to a certain haplotype, which has been associated also with coronary artery disease [18]. Additionally, there is evidence for Y chromosome-linked dysregulation of the lipid metabolism [19,20]. However, more studies are necessary to unveil the relevance of Y chromosome-encoded genes in these cardiovascular risk factors.

Pathophysiology of CAD differs between men and women, who express a pathological pattern with a characteristic microvascular dysfunction, which in turn is associated to an ischemic heart disease. Apart from the potential relation with chromosomes, there has been also determined polymorphisms of the genes involved in regulatory mechanisms of coronary blood flow [21,22]. In this regard, the protective role of genetic variants for ATP-sensitive potassium channel (KATP) in patients have been related to the susceptibility to ischemic heart disease and heart failure through microvascular dysfunction, in particular in females. Therefore, beside epigenetics, single nucleotide polymorphisms (SNP) offer new perspectives in the mechanisms underlying cardiovascular sex differences.

Gene expression responses to ischemia are different between females and males undergoing valve replacement, albeit the mechanism behind these differences has not been unraveled [23]. One of the genes with lower expression levels in females compared to males was again CMA1, previously mentioned in this chapter. In other patient cohorts, expression levels of the brain natriuretic peptide (commonly used as heart failure biomarker) are especially high in aged females [24]. Other metabolic genes, present also sexual dimorphism with a higher expression in females, which may confer certain protection in the onset of chronic pathological states like heart failure [25].

**Figure 2:**
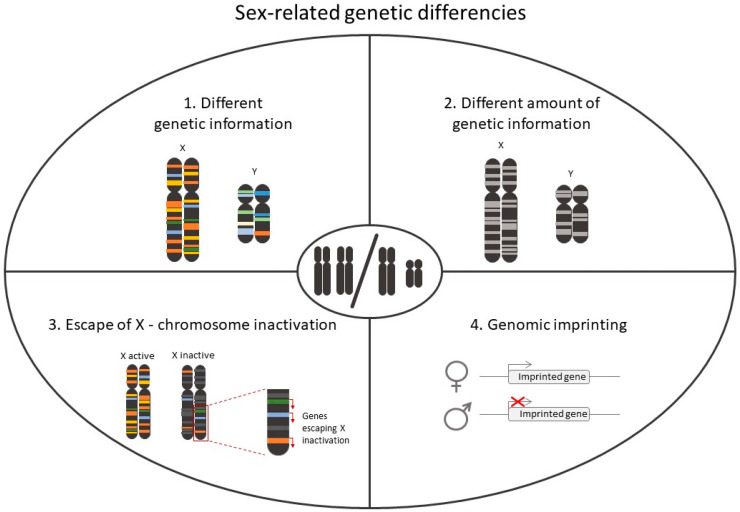
Representation of the main genetic differences in mammalian sex chromosomes.

Overall, genetics greatly affect disease responses and explain some sex-related differences (Figure 2). Hence, it is critical to take sex into account in the design of any animal and clinical studies having a translational potential. Personalization of healthcare can only be achieved in a sex-dependent manner. 

### 2.2. Role of Hormonal Status in Sex-Differences in CVD 

The largest cardiovascular risk factor that influences the development of CVD and the main factor that determines the differences on incidence between sexes is aging. Aging is associated with wholesale changes in physiology. Heart failure and stroke are two classical examples, with a higher risk in young males compared to females, but shifting to a higher incidence for female individuals after menopause [2,3]. Overall, early menopause as well as early age at menarche are associated with a 32% and 22% higher cardiovascular risk, respectively [26]. In middle-aged females (ages 45–55 years), the decline in estrogen levels leads to menopause and subsequent loss of fertility. This lack of estrogens (that is closely linked to aging) is related with the incidence of CVD and, aside from aging, the reason why the CVD appear in females an average of 10 years later than males [27].

Hormonal status is a main source of cardiovascular differences [28]. The most clear and obvious example studied is post-menopausal females, in whom the rapid decline of estrogens is correlated with an increment in CVD [29]. However, studies on the impact of hormonal replacement therapies (HRT) in postmenopausal females have yielded controversial and conflicting results [30]. The Women’s Health Initiative trials, designed to determine risks and benefits of HRT for chronic disease prevention in healthy postmenopausal females, reported no overall benefit on coronary heart disease risk [31]. However, subsequent analyses and follow-up of this trial demonstrated cardiovascular protective effects of estrogens in females initiating hormone therapy less than 10 years after the onset of menopause [31,32]. This fact is referred to as the “timing hypothesis,” and assumes the beneficial cardiovascular effects of hormonal replacement may occur when therapy is initiated before the negative effects of aging have become established, and could be related to a differential expression of estrogen receptors (ER) which modulates cardiovascular function [33,34].

### 2.3. Epigenetics in Sex-Differences in CVD

The genetic code represents one layer of biological information on an individual. The genome is relatively stable through a chronological (aging) and biological (cell divisions) lifetime and is the same in each and every cell of the body. The dynamic usage of the genome is the epigenetics, which defines how the genome is used in any given cell/nucleus at any one point in time. 

In essence, gene expression is regulated by epigenetic mechanisms, which are emerging as key regulators of differentiation, maturation, homeostasis, aging, and disease. In accordance with the central dogma of molecular biology, cellular, tissue, and organ function is governed entirely by ncRNA and proteins, which is in turn controlled by epigenetic mechanisms [35]. Ergo, sex differences in CVD risk, etiology, and phenotype must originate in sex differences in epigenetic regulation. However, how and why this occurs is only starting to emerge with increased awareness of sex differences in CVDs. 

#### 2.3.1. DNA Methylation

DNA methylation is considered a more stable of the epigenetic modifications. It is the covalent attachment of a methyl group (−CH_3_) to the fifth carbon of cytosine and is laid down in development and is deposited by de novo DNA methyltransferases, DNMT3a and DNMT3b, to help define and stabilize the differentiated cell type transcriptome. As a general rule, DNA methylation in promoter regions represses the gene expression. The role of gene body methylation is a little more unclear but CpG hypermethylation in gene bodies has been associated with higher gene expression in many cell lines and tissues [36,37]. Relevant and functional changes in DNA methylation patterns have been described in CVD in both human specimen and in pre-clinical experimental cardiology [38,39]. There are conflicting studies on the importance of DNMT3A/3B in pathological cardiac hypertrophy [40,41]. Components of the active demethylation pathway and hydroxymethylation marks are also emerging as regulators of gene expression in cardiac disease [42,43].

The methylation of genomic regions, far from being exclusive to the X-chromosome, also occurs in autosomal chromosomes contributing to genomic imprinting. Genomic imprinting describes the phenomenon when a gene is silenced depending on the parental origin of the allele. In humans, the list of imprinted genes includes approximately one hundred gene [44]. Genomic imprinting often depends on the presence of genomic sequences called insulators, which recruit certain protein factors blocking the transcription of upstream genes. The binding of the protein factors to the insulator regions can be disturbed by DNA methylation [45]. Noteworthy, imprinting genes have an organ-dependent regulation. A recent work with a well-studied imprinted gene, IGF2, showed an organ-dependent effect during fetal growth. However, the heart seems to develop similarly independently of sex [46].

DNA methylation abundance and patterns have been studied as markers of aging in different tissues, with the concept of a “Methylation clock” developed. Interestingly, the heart was one of the few organs whereby the methylation patterns did not significantly predict chronological age (*p* > 0.05 in test data sets) and male tissues in general exhibited accelerated aging according to the DNA methylation clock than females [47].

Because of a lack of comprehensive studies using both males and females with sex-specific analysis, the data on DNA methylation as underlying sex differences in the risk, types, and prognosis of CVD are in short supply. Overall underlying hypertrophy and disease-associated stimuli and gene expression changes have been broadly described as similar between sexes, perhaps suggesting little discrepancies in epigenetic mechanisms. However, a few deep mechanistic studies have identified different remodeling pathways and it is likely, in part because powerful (in terms of number and representation) pre-clinical and translational epigenetic studies have not been carried out, that no relevant dissimilarities have been identified. 

A few isolated and specific sex-specific DNA methylation patterns in CVD have been observed. Hypermethylation of the PLA2G7 gene promoter indicates higher risk of coronary heart disease in females only. PLA2G7 is a secreted enzyme that degrades platelet-activating factor [48].

The circulating neutrophil:lymphocyte ratio is emerging as a biomarker for inflammation and CVD. Unsurprisingly, lower blood cell Pentraxin 3 (PTX3) promoter DNA methylation levels were associated with higher PTX3 plasma levels and also neutrophil:lymphocyte ratio and presence of coronary artery disease. This phenomenon was only seen to a statistically significant level in males and not in females (*p* = 0.002 vs. 0.51) [49].

Another translational study into the usage of LINE-1 methylation in blood samples to assess risk and presence of cardiovascular and metabolic disease was performed on a population of adult Samoans, where 88 males and 267 females were examined. In this study, males had significantly higher LINE-1 methylation levels than females (*p* = 0.04) and elevated levels of LINE-1 methylation were associated with higher fasting LDL: HDL (*p* = 0.02). Thereby demonstrating the need for stratification of data according to sex for diagnostic and prognostic clinical research and application [50].

#### 2.3.2. Histone Modifications

Histone tail modifications as well as histone exchange are generally considered to be more dynamic than DNA methylation marks and are highly responsive to environmental cues such as signaling cascades, because of the relative steric accessibility of histone tails. The most intensely studied and understood marks are methylation and acetylation of H3 and H4 [51].

Histone acetylation profiling is used as a mark of open chromatin structure and active transcription, with clear and consistent associations between H3K4Ac, coinciding with low levels of DNA cytosine methylation, for example [52]. In the heart, it is well established that histone acetylation erasers, known as histone deacetylases (HDACSs) are responsive to calcium-centric canonical pro-hypertrophic cues such as calcium/calmodulin-dependent kinase II phosphorylation of HDAC4 as a key mechanism to initiate hypertrophic growth [53]. HDAC5 and HDAC9 knockout (KO) mice have exacerbated hypertrophic growth in response to genetic or pressure overload stress models [54]. Blocking Class II HDAC activation genetically or pharmacologically seriously blunts pathological hypertrophy and fetal gene activation [55]. Other triggers for HDAC activation in CVD include being responsive to intracellular lipid accumulation which can be seen in diabetes and obese patients, common comorbidities of heart failure [56,57]. The intricate integrated biochemistry of lipid-protein-transcriptome pathways in CVD are starting to be unraveled. 

Whole genome chromatin profiling in models of pathological hypertrophy and stress reveals wholesale changes in H3K9me2 that are also conserved in human. H3K9me2 writers GLP/G9a may be new targets for manipulation [58].

However, sex differences in histone modification profiles in the heart and vasculature have not yet been identified, while in other complex organs such as the brain, they are starting to be uncovered [59,60].

#### 2.3.3. RNA Modifications and Sex Differences 

DNA is not the only nucleic acid that can be extensively modified. RNA transcripts themselves undergo post-transcriptional modifications with more than 70 individual epitranscriptomic marks identified so far; the most well-studied being methylation of the 6th nitrogen of adenosine (m6A). As a newer area of investigation than epigenetic mechanisms on the nuclear genome, sex differences in health and diseases, novel writers, erasers, and readers of RNA modifications are still being identified in different species. Very few sex differences or sex-specific roles of the epitranscriptome have been described. One murine cytosine-5 RNA methyltransferase, NSun2, demonstrates male-specific infertility in KO mice [61]. A further example outside of the mammalian kingdom is that of the Arabidopsis ortholog of human mRNA adenosine methylase (MTA). Genetic ablation of MTA causes a loss of m6A and failure of the developing embryo to progress past the globular stage. MTA interacts with a homolog of the Drosophila Pre-mRNA-splicing regulator female-lethal(2)D, which is known to affect m6A deposition and mRNA splicing and is required for sex determination and its omission is enigmatically only lethal in females [62].

#### 2.3.4. X Chromosome Reactivation

As previously mentioned, in early embryogenesis, one of the X chromosomes in females is inactivated in an epigenetic process involving an antisense lncRNA (XIST) and the H3K27me3-writing polycomb repressor complex 2 (PRC2), gradually silencing one of the X chromosomes in a stable manner. DNA methylation marks are also in turn enriched on the inactive X. In this way, genes on the X chromosome are not necessarily more highly expressed or abundant in females than in males. However, there is an aging and degenerative disease link with X chromosome reactivation and re-expression of XIST and changes in H3K27me3 marks on the X chromosome have been observed in the general female population [63,64]. In particular, this phenomenon has been linked with autoimmune disease, with female autoimmune patients outnumbering males by a staggering 4:1 [65].

**Figure 3:**
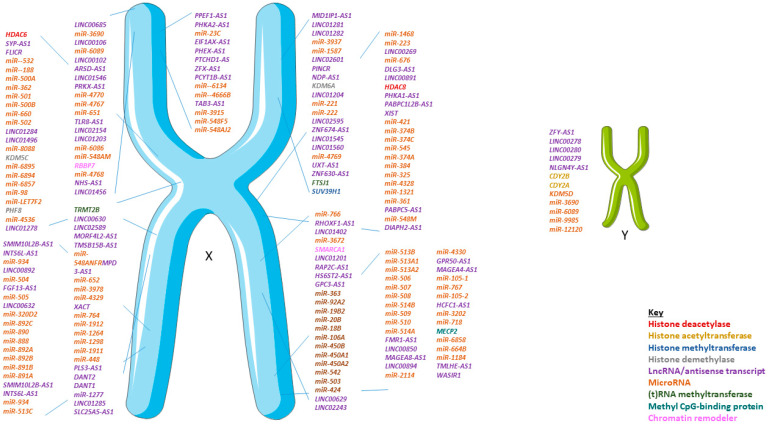
Epigenetic modifiers and non-coding RNAs encoded on the sex chromosomes in *H. sapiens.* Not to scale but the order indicates relative location on the genomic DNA.

Both the X and Y chromosomes encode a number of characterized and as yet uncharacterized epigenetic and epitranscriptomic modifiers and ncRNAs (Figure 3). Some epigenetic modifiers encoded on the X chromosome such as the relatively poorly characterized H3K27 demethylases KDM6A and KDM6B as well as the methyl-CpG-binding protein MeCP2. Conversely, KDM5D, is Y-chromosome encoded and is expressed in mouse and human hearts. Reactivation or overexpression of these KDM epigenetic erasers has not been investigated in health or disease. 

Other epigenetic and transcriptional regulators are sensitive to sex hormones themselves, with the activating histone acetylase (writer) complex p300/CREB-binding protein being responsive to activation of DNA by ER and androgen receptors (AR) [66]. In addition, the ATP-dependent chromatin remodeler BRG-1 is recruited by ERs in a cooperative manner to p300/CBP, coordinating histone acetylation with chromatin remodeling in a cooperative manner at the promoters of estrogen-responsive genes [67].

The study of abnormal X chromosome inactivation/reactivation is now emerging as another mechanism underlying sex differences in CVD. X-chromosome-encoded genes involved in post-translational modifications, epigenetic modifications, small-molecule biochemistry, and cell–cell signaling were more highly elevated in the blood of females than in males following cardiac ischemic events [68]. Given the effect seen on the female immune system, this concept could feature in underlying the elevated female risk for heart failure with preserved ejection fraction (HFpEF), with systemic and localized inflammation thought to be a key pathophysiological mechanism. 

As for other pre-clinical and translational studies, both male and female animal and cellular models as well as human specimen should be considered and sex-independent transcriptomic, epigenomic, and epitranscriptome analysis performed. Differences and changes could be diluted or uncovered when only analyzing one sex or combining both sexes in experimental cardiovascular research.

## 3. Non-Coding RNA and Sex Differences

While clear sex differences are documented in human disease, however relatively few studies have focused on searching for sex differences in ncRNA responses in human [69] (Table 1). NcRNA are separated into subfamilies attending the size, such as small-size microRNA (miRNA/miR, <200 nucleotides), lncRNA (>200 nucleotides), as well as circular RNA (circRNA). Both short RNA and lncRNAs show sex-specific patterns. 2754 lncRNA transcripts are encoded on the X-chromosome and 398 on the Y-chromosome according to LNCipedia in the high confidence dataset [70]. Hence, for discovery investigations focusing on ncRNA, it is essential to take into account sex. Sex disparities have been evidenced in pathologies like stroke, myocardial infarction (MI), and heart failure [71,72,73].

LncRNA are expressed and functional RNA do not encode proteins. In part because of their often poor conservation, low expression levels and highly spatial and temporally-specific expression profiles, lncRNA were only more recently discovered with the advent of deep RNA sequencing (RNA-seq). LncRNA can be encoded in a plethora of genomic contexts including antisense, intronic, intergenic (also knowns as long intergenic non-coding RNA) or bidirectional, the lncRNA promoter being within 1 kb (kilobases) of a protein-coding gene promoter and initiating transcription in the opposite direction [74].

While few have catalytic activity in isolation, lncRNA can act as cofactors or guides for proteins and in this way, may be regarded as epigenetic readers. As for protein coding genes, the biological roles of lncRNA span diverse functions, from direct gene expression and splicing regulation, miRNA sponges (affecting post-transcriptional processing), to acting as cofactors and mediators of for nuclear or cytoplasmic proteins, regulating membrane transport to cell–cell communication [74,75]. Differential expression of lncRNA in the cardiovascular system has been analyzed in both human specimen and animal models and a regulatory role for specific roles of lncRNA in CVD is established (Figure 3). Similarly for other studies in experimental molecular cardiology, many of these studies have either exclusively used males or not performed sex-stratified analysis of results. A systematic assessment of leading cardiovascular journals has reported that males were exclusively used in 71.6%, whereas females were exclusively used in 1.9%, while both sexes were included in 15.5% of 3396 analyzed cardiovascular studies [76]. Furthermore, male mice, rats, rabbits, or a combination of animal models are used in 95.0% studies [76]. A further systematic analysis focusing on sex differences in cardiovascular epigenetics identified that of the published translational cardiovascular studies describing only one sex, 86% studied males and 14% studied females only [77].

Epigenetic readers, writers, and erasers do not function in isolation but instead in co-operation. Often, an epigenetic reader or lncRNA may enable docking of an epigenetic writer or eraser together with a chromatin remodeling component, forming an active complex for epigenetic modification at a given locus at a given point in time. For example, the stress-associated BRG1-G9a-DNMT3 complex is formed in response to pro-hypertrophic stimuli, adding H3K9me2 and DNA cytosine methylation marks to the adult myosin heavy chain (MYH6) gene promoter [78]. Repression of MYH6 forms part of the reversion to fetal cardiomyocyte gene expression profile that is seen in pathological cardiac remodeling.

One example of a sex-differential epigenetic mechanism in CVD combines cooperation between an epigenetic reader (the methyl-CpG-binding protein MeCP2), a lncRNA (Mhrt), and a nascent miRNA transcript (miR-208b). Both Mhrt and pri-miR-208b are non-coding transcripts encoded within the cardiac myosin heavy chain gene (Myh7). Mhrt is protective against pathological remodeling by antagonizing BRG1 and preventing the upregulation of fetal myosin heavy chain (MYH7). MiR-208b is upregulated in female hearts in failing myocardium. The Mhrt methylation and MeCP2-dependent chromatinization of miR-208b in males attenuates elevation of miR-208b in health and disease compared with females [79].

A polymorphism in the locus encoding the lncRNA ANRIL seems to contribute to the increase of circulating levels of C-reactive protein [80]. ANRIL and another lncRNA named KCNQ1OT1 improved the prediction of left ventricular dysfunction after MI [81]. Interestingly, KCNQ1OT1 is encoded within an imprinting cluster of genes located in the chromosome 11 [82]. A recent study suggested a differential methylation in KCNQ1OT1 contributing to the differential risk of developing symptomatic long QT syndrome and it seems that the lncRNA is involved in the imprinting process [83]. One of the genes included in KCNQ1OT1 cluster, cyclin-dependent kinase inhibitor 1C (CDKN1C), was postulated as a novel female-specific biomarker of left ventricular dysfunction after MI [84]. This is certainly not an isolated case, as most imprinted clusters harbor both protein coding genes and ncRNA [85].

The most notable contribution of a lncRNA to sex differences is provided by the 17 kb (in human) lncRNA XIST, encoded on the chromosome X. This lncRNA contributes to the coating and subsequent random inactivation of one of the two X chromosomes in females [85,86]. The compensation of the genetic material is crucial, as many genes could be simultaneously expressed from both X-chromosomes, including the 118 miRNA encoded in X chromosomes. The Y chromosome encodes four miRNA (miR-3690-2, miR-6089-2, miR-9985, and miR-12120) and, although none of these have been shown to be associated to CVD yet, they could play a role in the regulation of inflammatory processes leading to disease development and outcome [87]. For example, it has been suggested that miR-6089 plays a role in inflammatory regulation in rheumatoid arthritis, by sponging TLR4 and NFκB—two players in cardiac inflammation [88,89].

As previously mentioned, X-inactivation is not a fully efficient mechanism, and this stands true for the miRNA as well. Indeed, 15% of the miRNA encoded by the X chromosome are able to escape X-inactivation [90,91]. Another phenomenon to take into account is the presence of duplicated copies of miRNA precursors throughout the genome. Interestingly, some of these replicas can be found in the sexual chromosomes, such as miR-92a, which has two predicted hairpin precursor sequences located either in the chromosome X or in chromosome 13 [87]. Such observation must be taken into consideration when drawing conclusions about RNA escaping X-inactivation. 

**Figure 4:**
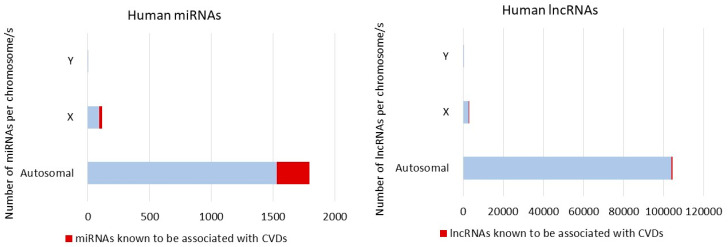
ncRNAs in CVDs. In light blue are the numbers of miRNAs (miRBase) and lncRNAs (LINCIpedia) encoded by autosomal, X- or Y-chromosomes. In red are the numbers of miRNAs and lncRNAs known to be associated with CVDs [70,87,100]. A literature search in association with matching analysis of ncRNAs present in the Heart Diseases related Noncoding RNA Database (HDncRNA—www.hdncrna.cardiacdev.com) have been used to generate these data [101].

Unlike the Y-linked miRNA, several miRNAs encoded by the X-chromosome appear to be deregulated in the context of CVD both in animal models and in human samples, as extensively reviewed elsewhere [69,92,93] (Figure 4). For example, miR-221 appears differentially expressed in metabolic syndrome only in women [94] potentially leading to cardiovascular disease onset. X-encoded miR-223, which has been associated with inflammatory processes and type 2 diabetes, regulates the expression of the glucose receptor Glut4 in cardiomyocytes [95]. In animal models of MI, inhibition of X-encoded miR-92 improved cardiac function through a reduced mortality of cardiomyocytes [96]. MiR-92 also regulates angiogenesis in animal models of ischemia [97]. However, it will be interesting to determine whether the X-encoded miR-223 and miR-92 present sex-specific phenotypes as the mentioned studies were not design to test sex-differences. On the other hand, the miR-221/222 cluster allows profibrotic signaling in murine hearts contributing to the complications associated with heart failure [98]. Interestingly, estrogen was reported to upregulate the miR-17-92 cluster among others and miR-221/222 can regulate, by direct or indirect targeting, the levels of ER [99]. It has been recently suggested that sex-biased miRNA may work as the underlying mediators leading to the sex-specific pathophysiology observed in females with HFpEF [93].

Other miRNAs not encoded by the X-chromosome show distinct sex-specific expression patterns (Table 1). Following stroke, circulating levels of miR-15a, miR-19b, miR-32, miR-136, and miR-199a-3p are elevated in aged females rats in contrast to males [102]. In the context of MI, miR-574-5p was associated with neurological outcome in women, but not in men conferring to this miRNA an added value as a potential new biomarker [103]. Several miRNAs present with sexual dimorphic expression in normal human hearts and a dozen arose in MI heart samples [104]. The miRNA profile of patients with coronary artery calcification was different between males and females, as studied in a cohort of patients with atrial fibrillation [105].

As for miRNAs, evidence is now emerging for sex differences in lncRNA expression and function in CVDs, which a large number of uncharacterized lncRNAs also encoded on the X chromosome. A polymorphism in the locus encoding the lncRNA ANRIL contributes to the raising of circulating levels of C-reactive protein [80]. ANRIL and another lncRNA named KCNQ1OT1 was found to improve the prediction of left ventricular dysfunction after MI [106]. Interestingly, KCNQ1OT1 is encoded within an imprinting cluster of genes located in the chromosome 11 [82]. A recent study suggested a differential methylation in KCNQ1OT1 contributing to the differential risk of developing symptomatic long QT syndrome and it seems that the lncRNA is involved in the imprinting process [83]. One of the genes included in the KCNQ1OT1 imprinted cluster, cyclin-dependent kinase inhibitor 1C (CDKN1C), was postulated as a novel female-specific biomarker of left ventricular dysfunction after myocardial infarction [84]. This is not an isolated case, as most imprinted clusters harbor both protein coding genes and ncRNAs [85]. Further research is needed to elucidate the precise contribution of ncRNAs to the differences in susceptibility, prognosis, and treatment response to different cardiovascular diseases observed in males and females.

## 4. Experimental Models to Study Sex-Related ncRNA Differences in CVD

Both in society and in the scientific community there is growing interest in the differences between males and females at different stages of life, as they affect the physiology and pathophysiology in health and disease as well as response to therapy. However, methodological considerations represent a serious barrier to research accounting for sex differences in CVD. The approach of these studies should include divergent sections such as the choice of the experimental animals and the most suitable biological control, the effect of sex hormones, the data analysis, and evaluation of experimental costs [107].

### 4.1. Including Both Sexes in CVD Models Will Refine RNA Research

Despite evident progress in inclusion of females in research studies in the last 20 years, most pre-clinical cardiovascular research is still primarily done using male animal models and male-derived cells resulting in incomplete and sex-biased data [108,109,110]. Preferential use of male animal models in basic cardiovascular research can be explained by higher variability in fluctuating gonadal hormone levels which request female testing across the estrus cycle, as well as sample size and costs implications of planning sex-based analyses [111]. In mice, estrogens are generally protective in various models of cardiovascular and associated diseases. Male and female animals differ in cardiovascular physiology and disease, independent of the type of gonadal hormones [4]. The argument that females are more variable because of estrus cycle has been questioned and indeed females are less variable than males as shown by the meta-analysis performed [112].

Variability in data may be increased when male and female are mixed. Recently, the international differences in the use of soy due to the presence of phytoestrogens in experimental diets have sex specific effects on expression of cardiac pathology [113].

Inbred mouse strains are valuable for varying sex and other variables when genetic background is held constant. Mice and humans differ in lipid profile, with mice carrying the majority of blood cholesterol in HDL particles, whereas humans have much greater levels of LDL cholesterol [114].

Sex and estrogen exert multitude pathways and cellular functions in the cells of cardiovascular origin. To date using animal models has allowed elucidation of specific effects of sex hormones on cardiac function including direct and indirect modulation of contractility, ion channel expression and function, reactive oxygen species production, and substrate use [115]. However, it is unclear how these pathways are regulated at the transcriptomic level.

### 4.2. Animal Models in Translational Cardiovascular Research

Although sex differences in cardiac gene expression have been documented in both humans and rodents, the study of sexual dimorphism in the ncRNA landscape in CVD remains largely unexplored representing a key knowledge gap. Sex difference in transcriptomic regulation could stem primarily from sex chromosome differences and genomic imprinting, and secondarily from epigenetic modifications such as DNA methylation, ncRNA-mediated regulation, and histone modifications, and direct and indirect effects of sex hormone [116]. By integration of existing mRNA microarray data from the human and mouse heart, sexual dimorphic gene have been identified in different cardiovascular pathologies including hypertension, ischemic cardiomyopathy, hypertrophic cardiomyopathy, heart failure and dilated cardiomyopathy [115].

Sexual dimorphic miRNA expression has been identified by microarray of miRNA isolated from mouse, human ischemic cardiomyopathy and normal heart samples [104]. Comprehensive RNA-seq revealed that the mRNA and lncRNA expression profiles in both atria and ventricles are distinct in males and females [117]. Currently, most transcriptomic cardiovascular studies focus on specific miRNAs or lncRNAs in one cell or tissues type in male animal or human specimen. Since sex-biased expression of ncRNA in cardiovascular system is poorly explored, an unmet need is to search for ncRNA regulatory mechanisms in the cell or cellular compartments as well as hormone-driven pathways related to sex specific cardiovascular pathology. 

Systematic analysis of transcriptomics data sets has demonstrated sexual dimorphism in expression profile of miRNA across different cell types and tissues. A recent analysis analyzed sex differences in miRNA expression and identified 73 female-biased miRNA and 163 male-biased miRNA across four tissues including brain, colorectal mucosa, peripheral blood, and cord blood (reviewed in [118]). These female miRNA and male miRNA exhibited higher evolutionary rate, higher expression tissue specificity, and lower disease spectrum width. Female miRNA are significantly associated with metabolism process and cell cycle process, and male miRNA tend to be enriched for functions like histone modification and circadian rhythm.

Transcriptome and functional profile of cardiomyocytes is also influenced by biological sex. Contractility was analyzed in the whole rat heart, adult rat ventricular myocytes (ARVMs), and myofibrils from both sexes of rats and functional sex differences were observed at all levels. Hearts and ARVMs from female rats displayed greater fractional shortening than males, and female ARVMs and myofibrils took longer to relax. An RNA-seq experiment performed on ARVMs from male and female rats identified ≈600 genes expressed in a sexually dimorphic manner, particularly through the protein kinase A pathway [119].

Sex dimorphism in cell response could be at least in part accounted for by sex-biased expression of regulatory elements such as miRNA. They took advantage of prior knowledge on specialized databases to identify X chromosome-encoded miRNA potentially escaping X chromosome inactivation (XCI). MiR-548am-5p emerged as potentially XCI escaper and was experimentally verified to be significantly upregulated in human XX primary dermal fibroblasts compared to XY ones [120].

Recently, circulating levels of three miRNA (miR-21-5p, miR-23a-3p, and miR-222-3p) and their target manganese superoxide dismutase (SOD2) were associated with high left ventricular post-MI in REVE-2 patients [121]. Interestingly, by analyzing separately females and males in this population, it was observed that the increase in circulating levels of miRNA was only significant in males whereas the circulating levels of SOD2 were only increased significantly in females. These results were supported by previous data showing that estradiol treatment significantly increases the expression of SOD2 both in mice and in human aorta endothelial cells leading to a decrease in oxidative stress [122].

The steroid hormone 17-β-estradiol and its receptors ERα and ERβ play important role in the development of sex differences in CVD [123]. Recent findings have demonstrated that ERβ may contribute to significantly altered expression of cardiac miRNA, for instance downregulation of miR-143 [123]. A widely expressed miR-181a regulates ERα in female cortical astrocytes and demonstrated protective effect against cerebral artery occlusion in female mice [124]. Circulating miR-181a has also been proposed as a biomarker for acute MI [124]. However, it has not yet been elucidated yet how ERα and ERβ might be regulated by miRNA in a sex-specific manner. Thus, further research on the role of E2/ER axis in sex specific cardiac miRNA expression including both females and males are required.

On the other hand, many questions regarding sex difference in their expression profile, relationship with disease progression in both female and males, biomarker value in disease prediction, and clinical outcome need to be addressed. In line with that including females and males in CVD models is mandatory for comprehensive understanding of ncRNA regulatory network in cardiovascular pathology underlying the sexual dimorphism, which in the end may lead to the improvement of clinical outcome by developing sex-specific diagnostic and therapeutic guidelines. Despite of potential challenges, miRNA therapeutics are processed in preclinical and clinical trial for treatment of several disease such as cancer and hepatitis C [125].

Mitochondrial metabolism is tightly related to cardiac function and sex differences in mitochondrial function in the heart have been observed in both humans and rodents [126]. In fact, the mitochondrial genome also contains hormone-responsive elements for estrogens and androgens [127]. ERα and ERβ are located in the mitochondria in cardiovascular cells, thus estrogen also modulates mitochondrial function in vascular and cardiac protection [128]. The location of ER in mitochondria is tissue-specific, being ERβ highly present in mitochondria of most tissues, such as skeletal muscle tissues, endothelial cells and cardiomyocytes, and mitochondrial ERα is predominant in the uterus, ovaries, and some breast cancer cells [129,130]. ARs have also been found in the mitochondria in human sperm cells and in some human prostate adenocarcinoma cell lines, although mitochondrial localization of ARs has not been extensively studied [131].

A number of proteins involved in mitochondrial metabolism are regulated in a sex-specific manner and targeted by sex-specific miRNA regulation. Mitochondrial dysfunction may result in unique transcriptional landscape, leading to impaired cardiovascular function, in which dysregulated ncRNA encoded either by nuclear or mitochondrial genome may play critical role [132]. Including both females and males in the investigation of mitochondrial ncRNA regulatory network in sex-specific mitochondrial dysfunction may lead to opportunity of development of improved and tailored diagnostic and therapeutic approaches for mitochondrial dysfunction-related CVD. 

Accordingly, dissecting the differences in ncRNA expression profiles between females and males is critical for precision medicine. A plethora of ncRNA found in plasma and blood samples have been identified as potential biomarkers for CVD [133,134,135]. 

Because of incomplete information on miRNA regulation independently in female and male cardiovascular systems in the setting of disease, miRNA-based therapeutics have not yet reached clinical trials for CVD. Promising results have been reported in numerous animal models of related diseases such as heart failure, cardiac hypertrophy, fibrosis, and hyperlipidemia [136,137]. In conclusion, including both males and female individuals and cells in ncRNA CVD research could pave the way for discovery of a novel and improved tailored generation RNA-based drugs for females and males.

### 4.3. Need for Adequate Experimental Models

Although there is no expectation that rodent animals and other experimental model organisms are identical to human, it is the main strategy for formulating basic biological concepts that help our understanding of human physiology. Animal models are essential tools to aim in understanding the origin and development of CVD and provide insight into mechanisms behind sex differences in heart diseases. The animal model of choice should contain a series of minimum requirements, such as the ability to be a model with hormone deficiency, to have genetic homogeneity, low cost, ease of experimental manipulation, and adequate ethical research principles. Rodents meet these requirements and are the first option in the research of sex differences in CVD research, researching sex dimorphisms in CVD [138].

An adequate experimental model is required because sex and estrogen-dependent mechanisms affect several organs in CVD. Knowledge on sex specificity in animal models on different signaling pathways and physiology is needed for understanding human diseases. A high number of studies favored male animals and this bias occurred in the majority of transgenic mouse strains with cardiovascular phenotype where significant sex differences are obvious [139].

Sex differences impact understanding of physiology, physiopathology, and response to therapy. Investigation of animals offers several advantages: diverse sex factors can be independently manipulated (including hormones and specific sex chromosome genes) to judge their separate effects, as well their interactions. It offers the ability to discover downstream molecular mechanisms, controlled by sex factors, which might be targets for therapy.

Investigations of rodent models in which causal factors can be isolated and studied in a more controlled manner will help to decipher the sex specific differences. Rodents are the most common species used to study cardiovascular function and diseases. The most commonly studied sex is male in both mice and rats.

In rodent primary cell cultures, sex was reported in less than 30% of studies, with 68.9% using exclusively male cells and none used solely female cells [110]. Mouse models are also used to discriminate hormonal and sex differential effects that cause sex differences. The aforementioned FCG and XY* mice have been described for measuring sex chromosome effect [4].

## 5. Mouse and Cell Experimental Models to Address Sex Differences in Epigenetics

### 5.1. Mouse Models

As mentioned above, translational as well as pre-clinical studies on rodent models have demonstrated that ncRNAs are differentially expressed and play a role in the pathophysiology of CVD. For instance, novel heart-specific lncRNAs involved in cardiogenesis and cardiac remodeling have been identified in a male-mice model of myocardial [140]. To define an adequate experimental model to study and advance understanding of sex differences in transcriptomics and epigenetics, it is important to consider the genomic and hormonal impact on cardiovascular pathophysiology.

Gonadal hormone impact in the pathophysiology of CVD has been studied in female and male rodent models by gonadectomy approaches. This surgical intervention often reverses the effect of sex differences in experimental animals, revealing that sex differences in CVD are largely controlled by sex hormone levels [141]. Depending on the objective of the study, it is important to consider the appropriate time points. It may be necessary to wait days or even weeks to visualize the effects of the absence of gonads as well as the effects of replacement therapy [107,142]. To define the effects of gonadal hormones it is usually necessary to perform an experimental design in at least three groups of animals: gonadectomized receiving placebo, gonadectomized receiving hormone replacement therapy and sham-operated group as scientific control. It is also important to take into account the plasma concentrations of sex hormones throughout life for the preparation of adequate hormone replacement therapy [143]. Physiological hormone contractions vary throughout life, more evident in females in whom plasma concentrations of 17 β-estradiol, the most active estrogen at cardiovascular level, have variations during the ovarian cycle in their fertile stage and abruptly decline in menopause.

In most mammals, lifespan does not exceed the reproductive capacity and they are usually fertile throughout their lives. However, the life expectancy in females is much higher than their reproductive capacity and around the middle age, the sudden drop in estrogen marks menopause. Thus, the surgical manipulation of rodents offers an effective alternative to study the gonadal effects in multiple systems, including cardiovascular [144]. Therefore, in order to mimic the hormonal status present in post-menopausal females and to understand sex differences in CVD, female rodents are usually subjected to ovariectomy (OVX) [145]. The OVX procedure consists of a clinical menopause induction by the excision of the ovaries and a subsequent depletion of ovarian hormones. The most appropriated surgical procedure in rodents is the dorsal procedure as the access to ovaries is easier and the surgery and recovery less harmful and faster than the other methods [144,146]. In sham animals, the ovaries are exposed but not removed. OVX animals are commonly used to evaluate the impact of natural and synthetic estrogens, and subsequently, exogenous hormones therapies (e.g., 17 β-estradiol and/or progesterone) can be administered to evaluate their effects on cardiovascular system [146].

The success of OVX is verified through body and uterine weight, hormonal profile, and cessation for the estrus cycle. Uterotrophic activity is confirmed through monitoring body weight and uterine dry weight [147,148]. Rodents experience a body gain and a loss of the uterine weight, which correspond to a diestrus phase associated with the decrease plasma levels of estradiol and an increase in luteinizing hormone (LH) and follicle stimulating hormone (FSH) levels [147]. However, monitoring sex hormones levels from the low steroid concentrations and low serum volumes of rodents, especially in mouse models, is difficult to achieve with immunoassays used for human samples. Unlike humans, rodents lack circulating sex hormone binding globulin (SHGB), contributing to a challenge of detection [149]. For these reasons, the high sensitivity and specificity of LC-MS/MS or GC-MS/MS assays have been used to analyze estradiol and other steroid levels from rodent serum and gonadal tissues in a single run [143,150]. Finally, vaginal smears demonstrates that the estrus regular cycle, that varies in 2 to 7 days in mice, changes after OVX and disappears in one week [115,151]. ER expression is also altered in a tissue-specific manner in ovariectomized rats, being increased the levels of ERα in the uterus, kidney, and cerebral cortex, but unaltered in the liver, cerebellum, heart, and thoracic and abdominal aorta [152].

However, the OVX model of experimental menopause has some limitations. First, it does not model the gradual natural transition in ovarian steroids experienced by humans (perimenopause) [153]. An alternative mouse model of menopause and aging has been proposed to emulate the natural events of perimenopause. Repeated injections of 4-vinylcyclohexene diepoxide induce a gradual depletion of primary and primordial follicles of the ovary. This transition is characterized by the fluctuation in estrogen quantity until low levels are reached and the rise in FSH levels, reproducing natural progression to human menopause [154]. Second, the age at which OVX is induced. In females, menopause occurs around the age of 50, when the effects of aging at vascular level begin to be evident. Therefore, an experimental model of menopause was needed in sexually mature animals, whose age corresponds to the middle age of females. In this regard, in the OVX model of senescence-accelerated mouse SAMR1/SAMP8 offer the advantage of separating the effects due to vascular aging from those due to the absence of estrogen [148]. Although surgical menopause in rodents induces more notable and sudden symptoms than that experienced by natural menopause, it is enough to observe changes in heart function and structure. 

Changes in the miRNA expression profile in cardiac structures from OVX rodents have been described. For instance, OVX in rats has been related with a downregulation of cardiac expression of miR-133 and miR-29, affecting their associated genes Bcl-2 and IGF-1, with anti-apoptosis and anti-fibrosis function [155]. OVX has also been useful in highlighting the role of miR-23a in cardiac disorders associated to estradiol deficiency. The increased levels of miRNA-23a reached in OVX induce the repression of connexin 43 in cardiomyocytes, which impairs cardiac gap junctions [156].

In males, testicles removal or orchiectomy (ORX) is also performed to study gonadal hormones impact on CVD. Surgical procedure is often realized when animals reach the sexual maturity (7–8 weeks and 2.5 months for mouse and rat, respectively). However in some experimental designs using AR KO rodents, mice are operated before puberty, at the age of 23–25 days and then supplemented with testosterone to reach physiological hormone levels [157,158]. ORX results in a reduction of body weight, circulating testosterone levels, vascular AR expression, and seminal vesicle weight [159]. 

After ORX, transcriptome profile changes. In rats, ORX decreases miR-132, which acts as a pro-angiogenic factor playing a role in CVD, and the testosterone replacement therapy improves the heart angiogenesis by increasing miR-132 in diabetic male rats [160].

Sex hormones mediate biological actions in part through hormone receptors. Another approach to evaluate implications of specific steroid receptors is through murine genetic manipulation. In this regard, nuclear ER KO mice ERα-/- and ERβ-/- have made possible the assessment of their different physiological roles and ligand-binding domains [161]. ERα KO mice (ERα-/-) are infertile and have severely weakened spermatogenesis [162]. Two miRNAs, miR-100 and Let-7b, which target the ERα gene, have been associated with infertility, establishing a link between post-transcriptional gene regulation and the ER [163,164]. There are also studies with cardiac-specific omission of ERα to describe the effects of this receptor subtype specifically in the heart, and to determine the role of ERα on gene expression in cardiomyocytes [165]. ERβ KO mice (ERβ-/-) have also been used to demonstrate the modulation by ERβ of vascular relaxation to estradiol [166]. Likewise, other pathways such as that responsible for the acute estrogen-dependent vasodilation mediated by nitric oxide and blood pressure-lowering activity has been established by using G protein-coupled ER (GPER) KO mice [167]. Overall, KO mice are useful to study the effects of steroids hormones on specific tissues, including cardiovascular.

Genetic models in experimental animals have also been generated to determine membrane or intracellular mechanism of ER action. For instance, ERαAF-2° mice model does not show estradiol nuclear functions as it lacks AF-2, the region of transcriptional activation located at the E domain of both ER, but maintains membrane effects [168]. On the contrary, C451A-ERα mouse model, or NOER (nuclear only ER), shows a mutation of the ERo palmitoylation site, which is crucial for ER and caveolin-1 association at the plasma membrane and lacks some estradiol actions in vasculature such as the phosphorylation of endothelial NO synthase (eNOS) and subsequent vasorelaxation [169]. These in vivo approaches help to demonstrate specific roles for membrane and nuclear ER in physiological processes and pathologies.

#### The “Four Core Genotypes” Model

To study the effects of the genetic component over and above the influence of sex hormones, a mouse model system that offers some advantages to study sex differences has been developed. It is the “four core genotype” (FCG) mouse model that is becoming a standard in the research of sexual differences. In the FCG model, the sex chromosome complement, XX or XY, is independent of the gonadal hormones. The model comprises XX and XY gonadal males, and XX and XY gonadal females, and the effects attributed to genetic background can be easily separated from those of gonadal hormones [170]. In CVD, FCG mouse model has been used for instance to demonstrate the relationship of ischemic stroke sensitive with gonadal hormones rather than sex chromosomal component and proving that stroke outcomes worsen in XX genotype compared to XY chromosomes [171].

### 5.2. Vascular Cell Cultures

In experimental research with both sexes represented, in vitro cellular models are not an exception. Sex can have a marked impact on the biology of cells as has been contrasted in primary cultures of human umbilical vein endothelial cells (HUVECs) where sex influences multiple phenotypic and functional characteristics [172]. However, although more and more academic journals have a policy that the source of cells utilized should be clearly indicated when submitting an article for publication, there are still very few studies that consider the sex of the cultured cells [173]. In experimental design, it is as important to use cells from both sexes, as it is to stratify the results by sex.

To verify the sex of the cells, the most suitable alternative is the use of molecular genetics commonly used in a cultured cell research laboratory, such as polymerase chain reaction, PCR-based assay. Many Y-chromosome specific markers could be useful in sex determination biological samples, showing sex-specific sequence and size divergences. For instance, amelogenin gene, codified by AMELX and AMELY in X- and Y chromosome respectively, is commonly used in forensics although with various kinds of discrepancies associated with ethnicities or PCR inhibitors [174]. The magnitude of variation of steroid sulfatase gene (STS) is sometimes used to differentiate STS homologue on X and Y chromosome [174]. In basic research, rather than focus on the degree of variation in expression certain amplicons, the gold standard could be the test of a gene or fragment only expressed in a unique sex on the Y chromosome, giving us a rapid and clear answer to the sex of the studied cells. The SRY gene—sex determining region Y—which promotes the male sexual development is only located on Y-chromosome, and has shown its potential as sex-marker. Through a PCR, sex of the cells is determined, being male by the presence, and female by the absence of the SRY gene [175]. There is also a possibility to use lncRNAs to determine the sex of certain body fluids as was shown for XIST and RPS4Y1 [176].

In addition to the chromosomal impact on cell biology, it is interesting to analyze the myriad of effects that sex hormones have on cells through signaling pathways. It is important to note that cardiovascular cells in culture express sex hormones receptors (reviewed in [177]). Because of the modulation of several biological functions such as gene expression and signaling pathways, the most important are the ER and AR.

Estrogen actions result from the activation of transcriptional and non-transcriptional signaling pathways through binding to different types of receptors. The classical and nuclear ERα and ERβ are located in the cytoplasm and once bound to estradiol form dimers, which translocate to nucleus and regulate gene expression directly by interacting with estrogen response element (ERE) and recruiting co-activators or co-repressors, or indirectly by associating with other transcription factors such as activator protein 1 and specificity protein-1 [178]. Most of the vascular protective effects described for estradiol are mediated by ERα particularly in endothelial cells [179]. Full ERα and ERβ, and truncated splice variants of ERα (ERα46 and ERα36) have been also located on plasma membrane cell, and besides GPER mediated rapid responses to estradiol [180]. Similarly, testosterone, the primary sex hormone in males, and its active metabolite 5α-dihydrotestosterone also show cardiovascular effects by binding cytosolic AR and triggering signal through both genomic and non-genomic mechanisms, involving rapid induction of second messenger signal transduction cascades [181]. Therefore, it is necessary to utilize specific tools to characterize the type of receptor involved in a particular vascular response, as well as methods to locate the receptor in the membrane or in the cytoplasm of the cell. In this sense, pharmacological agonists and antagonists of ER and AR are widely used both in cultured cells and in in vivo studies. Selective estrogen receptor modulators (SERM), synthetic ligands of ER, such as tamoxifen or raloxifene, can act both as agonists or antagonists in different tissues through modifying the ER function [182,183]. Among naturally occurring phytoestrogens, isoflavones such as genistein and daidzein can also act as agonist or antagonists in relation to endogen estrogens, and experimental studies have shown beneficial effects of isoflavones on endothelial and vascular smooth muscle cells [184].

Currently, identifying the ER location in the cell remains difficult, especially with immunochemistry or immunocytochemistry approaches, and there is little information in this regard. It has been demonstrated that nuclear or extra nuclear ER detection differs depending on the epitope of ER targeted by the antibody. For instance, antibodies targeting ER NH2-terminal sequence, ER21, interact with more nuclear ER than membrane ER, as opposed to antibodies against the COOH-terminal sequence, SRA1010 [185]. Further, discriminating ERα isoforms with antibodies, shows non-specific bands, is not the most reliable approach for immunofluorescence labeling assays [186]. However, antibodies directed to the unique C-terminal 27 amino acid tail of the membrane ERα36 isoform have been used in various types of cells to detect its location [187].

In vitro approaches have been developed to distinguish between non-genomic and genomic ER signaling actions. Agents such as bovine serum albumin, which forms a complex unable to cross the membrane, could hamper the E2-ER binding have been used to characterize ER-membrane actions [188]. Another membrane-impermeable estrogen conjugate is the estrogen dendrimer conjugate (EDC). Estrogen conjugated to positive charged non-degradable polyamidoamine (PAMAM) dendrimers have allowed to identify non-genomic actions of estrogens such as endothelial nitric oxide synthase (eNOS) activity, proliferation of endothelial cells, and re-endothelialization in carotid injury [189,190]. A further efficient approach to stimulate non-genomic signaling via specific activation of membrane is through pathway-preferential estrogen (PaPEs), a small molecule similar in structure to E2, which activates preferentially membrane receptors, and even has allowed associating an eNOS non-nuclear pathway with the repair of vascular endothelium in carotid artery injury [191].

It is also necessary to highlight some recommendations for cell culture media to study sex hormones. Many common components of culture media show estrogenic activity that may interfere with the studied sex hormones effects. For instance, fetal bovine serum (FBS) is widely added to culture media because it contains many non-defined growth-promoting factors and nutrients necessary for cell survival and proliferation, but is also a relevant and variable hormonal source to be avoided. Dextran-coated charcoal-stripped FBS is often used to avoid endogenous steroid and peptide hormones from the serum, improving reproducibility by reducing the impact of steroid hormones on cells [192,193]. Likewise, the pH indicator commonly present in most tissue culture media, phenol red, has a weak estrogen activity and can stimulate some estrogen-sensitive cells [194]. Thus, the estrogenic activity of phenol red should be considered in any studies that utilize estrogen-responsive cells [195].

## 6. Conclusions and Future Directions

In order to approach the study of sex differences in CVD and in epigenetics in CVD in particular, it is necessary to have basic knowledge that allows us to choose and apply an appropriate experimental design. In this review, we have tried to highlight the importance of sex-stratified analysis in data obtained from clinical and pre-clinical research as well as the tools we have to carry out fundamental research, both in animal and cellular models. With all this, from this COST action we encourage the entire scientific community to include sex in their experimental designs to determine its impact on the results obtained.

## Figures and Tables

**Table 1:** Sex-biased expression of miRNAs in CVDs. All chromosomal origins refer to human even when the study was conducted in other organisms. When the isoform of the miRNA was not specified in the reference, all the possible chromosomal origins have been annotated. In addition, when more than one isoform is present in the same row the chromosomal origin of each isoform is reported.

MiRNA Identifier	CVD	Specificity	Regulation	Chr	Organism Studied	References
miR-103	coronary artery calcification	♂	?	20–5	Human	PMID:30465521
miR-106a/b	cardiac fibrosis	♂	Hormonal	X–7	Mouse	PMID:24157234
miR-125a-5p	coronary artery calcification	♂	?	19	Human	PMID:30465521
miR-126	coronary artery calcification	♀	?	9	Human	PMID:30465521
miR-136	post-stroke	♀	?	14	Rat	PMID:24428837
miR-142-3p	postmenopausal HRT *	♀	Hormonal	17	Human, mouse	PMID:25040542
miR-15a	post-stroke	♀	?	13	Rat	PMID:24428837
miR-182	postmenopausal HRT *	♀	Hormonal	7	Human, mouse	PMID:25040542
miR-199a-3p	post-stroke	♀	?	1	Rat	PMID:24428837
miR-19b	post-stroke	♀	?	13–X	Rat	PMID:24428837
miR-212	descending aorta calcification	♀	?	17	Human	PMID:30465521
miR-221	coronary artery calcification/metabolic syndrome *	♂/♀	?	X	Human	PMID:30465521/ PMID:24093444
miR-222	insulin resistance in gestational diabetes mellitus *	♀	Hormonal	X	Human	PMID:24601884
miR-223	coronary artery calcification/postmenopausal HRT *	♂/♀	?/Hormonal	X	Human, mouse	PMID:30465521/ PMID:25040542
miR-23a	post-stroke/post-menopause related arrhythmia	♀	Apoptotic/Hormonal	19	Mouse/rat	PMID:21709246/ PMID:25798059
miR-24	cardiac fibrosis	♂	Hormonal	9–19	Mouse	PMID:24157234
miR-27a/b	coronary artery calcification/cardiac fibrosis	♀/♂	?/Hormonal	19–9	Human/mouse	PMID:30465521/ PMID:24157234
miR-32	post-stroke	♀	?	9	Rat	PMID:24428837
miR-34a	dilated cardiomyopathy	♀	?	1	Mouse	PMID:27270487
miR-363-3p	neuroprotective for stroke	♀	Apoptotic	X	Rat	PMID:27773791
miR-574-5p	cardiac arrest	♀	?	4	Human	PMID:31275442
miR-let7g	metabolic syndrome *	♀	?	X	Human	PMID:24093444

*: Conditions or diseases that are associated to CVDs. Abbreviations: CVDs (cardiovascular diseases), chr (chromosome), HRT (hormone replacement therapy). Symbols: ♀ = female; ♂ = male; ? = unknown.

## Abbreviations

AR	Androgen receptor
ARVM	Adult rat ventricular myocytes
CVD	Cardiovascular disease
eNOS	Endothelial nitric oxide
ER	Estrogen receptor
FCG	Four core genotype
FSH	Follicle stimulating hormone
HDAC	Histone deacetylase
HRT	Hormone replacement therapy
Kb	kilobase
LncRNA	Long non-coding RNA
MI	Myocardial infarction
miRNA/miR	MicroRNA
ncRNA	Non-coding RNA
ORX	Orchiectomy
OVX	Ovariectomy
RNA-seq	RNA-sequencing
SERM	Selective estrogen receptor modulators
SRY	Sex determining region Y
XCI	X chromosome inactivation
